# Land cover preferences and spatiotemporal associations of ungulates within a Scottish mammal community

**DOI:** 10.1002/ece3.11015

**Published:** 2024-02-09

**Authors:** Connor Lovell, Nathalie Pettorelli, Terence P. Dawson

**Affiliations:** ^1^ King's College London London UK; ^2^ Institute of Zoology Zoological Society of London London UK

**Keywords:** camera trapping, land cover, Scotland, spatiotemporal interactions, ungulates

## Abstract

In the degraded and modified environment of the Scottish Highlands, novel ungulate communities have arisen following local extinctions, reintroductions, and the introduction of non‐native species. An understanding of the dynamics and interactions within these unique mammal communities is important as many of these mammals represent keystone species with disproportionate impacts on the environment. Using a camera trap survey, we investigated land cover preferences and spatiotemporal interactions within a Scottish ungulate community: the sika deer (*Cervus nippon*), the roe deer (*Capreolus capreolus*), the red deer (*Cervus elaphus*), and the wild boar (*Sus scrofa*). We used generalised linear models to assess land cover preferences and the effect of human disturbance; spatiotemporal interactions were characterised using time interval modelling. We found that sika deer and roe deer preferred coniferous plantations and grasslands, with sika deer additionally preferring woodland. For red deer, we found a slight preference for wetland over woodland; however, the explained variance was low. Finally, wild boar preferred grassland and woodland and avoided coniferous plantations, heathland, and shrubland. Contrary to our expectations, we found no evidence that human disturbance negatively impacted ungulates' distributions, potentially because ungulates temporally avoid humans or because dense vegetation cover mitigates the impacts of humans on their distributions. Furthermore, we detected a spatiotemporal association between sika deer and roe deer. Although the underlying cause of this is unknown, we hypothesise that interactions such as grazing facilitation or an anti‐predator response to culling could be driving this pattern. Our work provides a preliminary analysis of the dynamics occurring within a novel ungulate community and also highlights current knowledge gaps in our understanding of the underlying mechanisms dictating the observed spatiotemporal associations.

## Graphical abstract

Using a camera trap survey, we investigated land cover preferences and spatiotemporal interactions within a Scottish ungulate community: the sika deer (*Cervus nippon*), the roe deer (*Capreolus capreolus*), the red deer (*Cervus elaphus*), and the wild boar (*Sus scrofa*). Ungulates showed some land cover preferences, whilst we found evidence of a spatiotemporal association between sika deer and roe deer. Although the underlying cause of this is unknown, we hypothesise that interactions such as grazing facilitation or an anti‐predator response to culling could be driving this pattern.

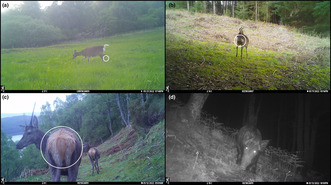

## 1 INTRODUCTION

The Scottish Highlands are a highly modified environment containing unique ecosystems and wildlife communities. Humans modified the Scottish environment considerably from as early as 11,000 BP through land clearance for agriculture (Dumayne‐Peaty, 1999; O'Sullivan, 1974; Smout et al., 2007). Much of Scotland's woodland was subsequently lost from the 1500s to the 1900s with humans as a significant driving force (Smout et al., 2007). Currently, most of the country is classified as rural, with major land uses including agriculture, forestry, hunting, and wildlife conservation (Brand, 2021; Morgan‐Davies & Waterhouse, 2010).

This extensive modification of the Scottish landscape, when coupled with hunting, led to the loss of many of Scotland's large keystone animals (species whose effects on the environment are large relative to their population size) such as the moose (*Alces alces*), auroch (*Bos primigenius*), Eurasian lynx (*Lynx lynx*), and grey wolf (*Canis lupus*; Bishop et al., 2015, Brown et al., 2011, Power et al., 1996, Warren, 2009). The remaining large terrestrial mammals are all ungulates, and include the native red deer (*Cervus elaphus*) and roe deer (*Capreolus capreolus*), non‐native sika deer (*Cervus nippon*) and fallow deer (*Dama dama*), and, more recently, wild boar (*Sus scrofa*), reintroduced to the British Isles by humans a few decades ago (NatureScot, 2016, 2022).

How ungulates in these novel communities behave and interact is important to understand as Scottish ungulates are often classed as keystone species and therefore have a significant influence over the environment through ecosystem processes such as browsing, grazing, and rooting (Power et al., 1996; Rooney, 2001; Sandom et al., 2013a). In addition, the lack of wild predators in Scotland leads to higher ungulate densities, with potentially increased competition and unusual behavioural interactions as a result (Latham, 1999; Simberloff, 1982). Finally, the presence of non‐native species in Scotland is likely to alter the behaviours of, and interactions between, ungulates as these species have not co‐evolved (Ferretti & Mori, 2020; Latham, 1999).

A previously studied factor influencing ungulates' distribution is land cover type (Braza & Alvarez, 1987; Uzal et al., 2013; Welch et al., 1990). Food availability and vegetation cover both influence ungulate habitat selection, with a trade‐off existing as high vegetation cover shades out high‐quality forage (Mayle, 1996; Mysterud & Østbye, 1999). The red deer is generally observed on open heathland, with some spring preference for grassland and winter preference for coniferous woodland and plantations (Putman, 1996; Schaefer et al., 2008; Ward, 2005). Red deer rarely occupy dense closed forests, instead preferring forests with open rides (long narrow glades) and clearings where heather and plant cover is higher (Mitchell et al., 1977; Welch et al., 1990). Having said this, some level of forest cover appears important for red deer (Borowik et al., 2013). Similarly, roe deer benefit from some forest cover (particularly deciduous and mixed forests) and other high‐cover dense vegetation types, but select heavily for areas with plants in the most nutritious phenological stage (Borowik et al., 2013; Mancinelli et al., 2015; Palmer & Truscott, 2003; Welch et al., 1990). Indeed, roe deer fawn winter body mass (a strong determinant of fitness) can be heavily influenced by individual plant species (Pettorelli, Dray, et al., 2003).

However, the distribution of other wild ungulate species in Scotland, such as the sika deer (introduced c.1860) and the wild boar, is less well understood (Ratcliffe, 1987; Swanson & Putman, 2009). Research conducted in Southern England suggests sika deer prefer coniferous plantations and heathland, and other vegetation types providing high cover and grazing potential (Mayle, 1996; Putman, 1996; Putman & Pemberton, 2022; Uzal et al., 2013). Similarly, research on sika deer in their native range finds a preference for deciduous, coniferous, and mixed forests (Sakuragi et al., 2002, 2003). A preference for shrublands and grasslands is also observed in their native range, particularly when the latter is located nearby forest cover (Honda, 2009; Laneng et al., 2023). Having said this, some studies record no preferences for sika deer (Borkowski & Furubayashi, 1998). For wild boar, the research conducted in Scotland to date suggests a preference for woodland, particularly deciduous woodland, and grassland (Sandom et al., 2013a). However, these were captive individuals and further research is required to determine whether they would remain close to woodlands if free roaming (Sandom et al., 2013a). In southwest England, recent work has identified forest as an important predictor of wild boar distributions, alongside open parkland (Bacigalupo et al., 2022). This is similar to mainland Europe, where multiple studies identify forest cover as an important predictor of wild boar distributions, with a preference for deciduous over coniferous forests often observed (Borowik et al., 2013; Jánoska et al., 2018; Thurfjell et al., 2009; Virgós, 2002). Some studies additionally demonstrated a preference for open areas such as pastures and meadows, although others fail to detect a preference for open areas (Bacigalupo et al., 2022; Jánoska et al., 2018; Thurfjell et al., 2009). As species vary in their preferences both within populations and geographically, it remains important to consider land cover selection in varying ecosystems (Alston et al., 2020; Shy, 1984).

One additional factor influencing ungulate spatial distributions is anthropogenic disturbance. Prior studies demonstrate a strong influence of human activities and disturbance on ungulate behaviour. Studies demonstrate how ungulates avoid human infrastructure such as roads, vehicle traffic, and dwellings (Bojarska et al., 2020; Bonnot et al., 2013; D'Amico et al., 2016; Ikeda, Kuninaga et al., 2019). However, the need for ungulates to use particular land cover types (such as woodlands) may also mask a negative response to humans (Wevers et al., 2020). Indeed, Wevers et al. (2020) found no relationship between human disturbance and roe deer habitat use, and a positive relationship between hunting high seats and wild boar activity. Although the latter is potentially due to hunters baiting high seats, in both cases selection for high‐cover vegetation types may modulate and reduce any negative influence of human disturbance.

A less studied process affecting ecological communities are non‐trophic interactions – interactions between species that are non‐consumptive (Kéfi et al., 2015; Majdi et al., 2014; Ohgushi, 2008). Non‐trophic interactions are traditionally classified based on their impacts on species, ranging from mutualism (positive effect on each species) to competition (negative impact on each species; Bronstein, 2015; Burkholder, 1952; Latham, 1999). Non‐trophic interactions can alter the spatiotemporal activity of animals: the presence of a mutualism between two species can lead to interspecific attraction between them, whilst competition could lead to two species showing interspecific avoidance (Asefa, 2016; Karanth et al., 2017). Therefore, animals potentially alter both where and when they are active in response to an interacting species (Cusack et al., 2017; Durant, 1998; Ferretti et al., 2011). Although inferring the underlying non‐trophic interaction from observed interspecific attraction and avoidance is difficult and the underlying behavioural and methodological processes need careful consideration, research has demonstrated the use of interspecific attraction and/or avoidance in identifying non‐trophic interactions (Cusack et al., 2017; Karanth et al., 2017; Niedballa et al., 2019). For example, Karanth et al. (2017) found evidence of interspecific competition through fine‐scale behavioural avoidance between carnivores, whilst Cusack et al. (2017) demonstrated, with varying success, how kleptoparasitism between African carnivores leads to interspecific attraction.

In Scotland, red deer appear to exert a competitive effect on roe deer. Studies demonstrate a negative effect of red deer density on roe deer numbers, with resource competition potentially causing a negative impact on roe deer body mass (Borkowski et al., 2021; Latham et al., 1997; Richard et al., 2010). With sika deer, some limited research demonstrates the potential for sika deer to be displaced by red deer, and for high red deer densities to reduce fertility in sika deer (Putman & Pemberton, 2022; Raymond, 2008). Interactions between sika deer and roe deer are poorly understood, and restricted to analysis of dietary overlap with no evidence of a competitive effect (Putman, 1996). Very little is known for wild boar interactions in Scotland, although in mainland Europe segregation is detected between roe deer and wild boar (Zanni et al., 2021). Subsequently, interactions within this unique ungulate community warrant further investigation.

This study assesses the springtime land cover preferences and spatiotemporal relationships within a novel Scottish large mammal community consisting of two native species (red deer and roe deer), a non‐native species (sika deer), and a reintroduced native species (wild boar). Springtime represents an important time for the ungulates present onsite. Individuals present will be feeding on new vegetation growth to maximise energy intake after harsher winters; with different species heavily pregnant, birthing, feeding young, and/or defending territories at this time of year (Gaillard et al., 1993; Johansson, 1996; McCullough et al., 2009; Pettorelli, Gaillard et al., 2003; Stopher et al., 2008).

We first investigate the role of land cover in influencing species' detection rates. We expect land cover that provides cover and more abundant forage resources (such as grasslands) to be preferred (Allwin & Swaminathan, 2016; Mayle, 1996; Meriggi & Sacchi, 2001). We also anticipate that human disturbance would negatively impact ungulate distributions. Following this, we investigate whether spatiotemporal associations are present between these ungulates, with the expectation that roe deer will avoid all other species spatiotemporally due to interspecific competition (Latham, 1999; Mori et al., 2020).

## 2 METHODS

### 2.1 Study area

The Bunloit rewilding project is a 511‐ha site in Inverness‐shire, part of Highlands Rewilding (https://www.highlandsrewilding.co.uk/). The project aims to use rewilding to maximise both biodiversity and carbon capture in the area. Vegetation types found onsite include mixed woodland (*Quercus* sp., *Betula* sp., *Pinus sylvestris*), coniferous plantations (*Picea sitchensis*, *Larix* sp., *P. sylvestris*), grassland, wetland, and heathland. Sika deer, roe deer, red deer, and wild boar, alongside other mammals such as the European badger (*Meles meles*), the red fox (*Vulpes vulpes*), the red squirrel (*Sciurus vulgaris*), and the pine marten (*Martes martes*), are all present onsite (Highlands Rewilding Ltd, 2021a). With regard to the wild boar, their origins are unknown as they were present at least 15 years prior to the creation of the Bunloit rewilding project (Scott Hendry, 2022). However, numerous wild boar farms recorded in the area would suggest they are escapees (Massei & Ward, 2022). Additionally, camera trap images from the project's baseline surveys would suggest they are a mix of wild boar and hybrid wild boar/feral pigs, like most UK wild boar populations (Alister Hughes‐Roden, personal communication, June 24, 2022).

### 2.2 Camera trapping

Camera traps were used as a cost‐effective, non‐invasive method able to conduct multi‐taxa surveys continuously at the landscape level (Caravaggi et al., 2017). The use of camera traps in ecological research, including in exploring land cover preferences and spatiotemporal associations, has grown over the last decade (O'Connell et al., 2011; Van Berkel, 2014). Crucially, for each detection of an animal, camera traps record both a spatial aspect (where the animal is located) and a temporal aspect (what time the animal was active at), therefore permitting spatiotemporal analyses to be conducted.

**FIGURE 1:**
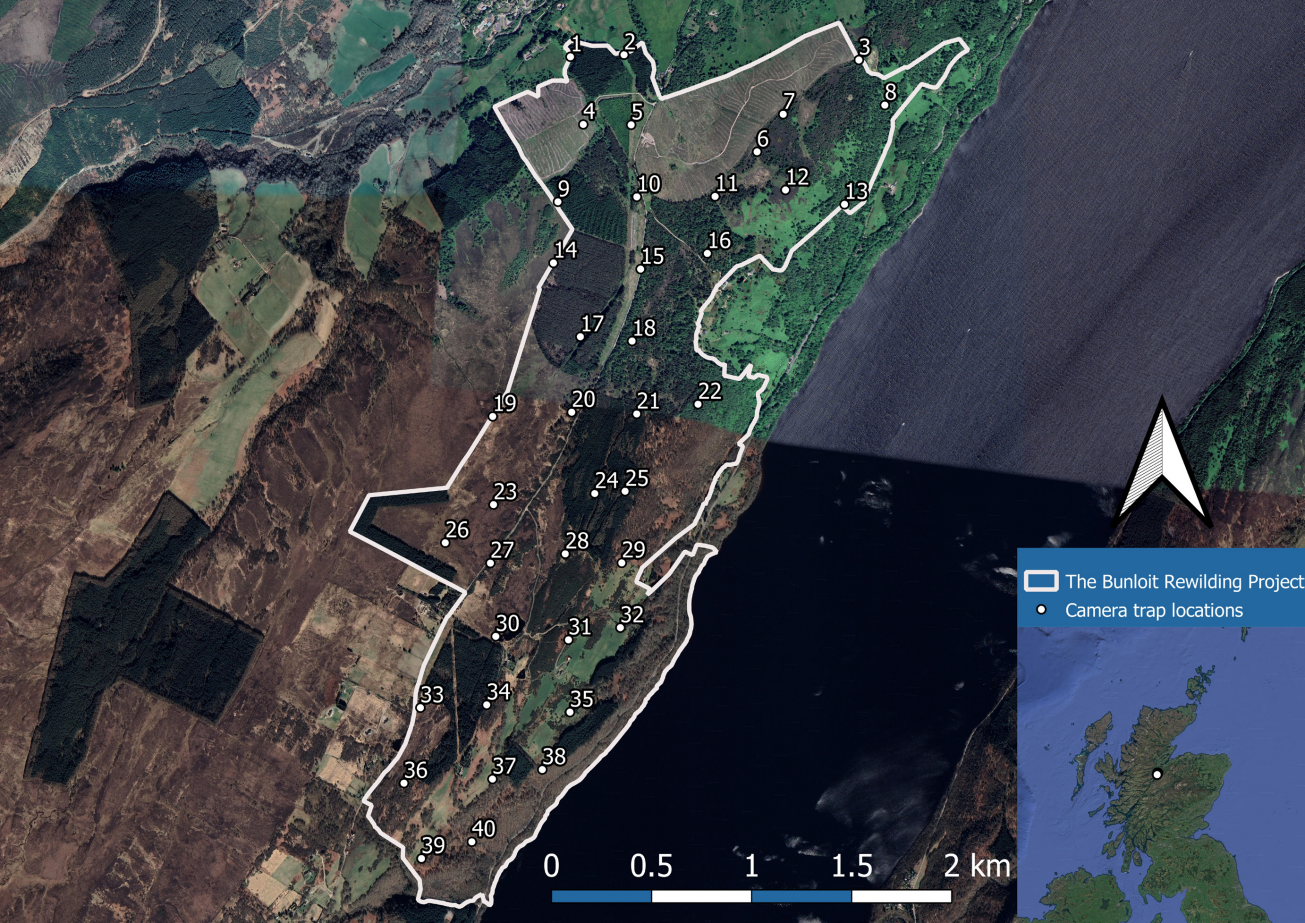
Camera trap placements across the Bunloit rewilding project. The insert illustrates the location of the site within Scotland. Map created with QGIS v.3.16.4.

We placed 40 camera traps across the Bunloit rewilding project using random systematic sampling. Within the boundary of the site, a grid with grid squares of size 364 m was generated from a randomly selected point using *Gridmaker* (Rowcliffe, 2022). Camera traps were then placed as close to gridline intersections as possible accounting for the ease of relocating camera traps, the structures present which cameras can be securely attached to, and both the practical and safety implications of the site (Figure 1; Van Berkel, 2014). These practicalities meant the shortest distance between two camera traps was approximately 152 m.

Cameras were deployed over the period 3 May 2022–7 May 2022 and were set to record for a 28‐day period. After 28 days, the cameras were collected in the same order they were deployed between 31 May 2022 and 4 June 2022. Deploying 40 camera traps for 28 days is identified as being sufficient to record the species present within a survey area, and is comparable in terms of camera trap‐days to similar studies on large mammals (Akbaba & Ayaş, 2012; Kays et al., 2020; Kelly & Holub, 2008).

All camera traps were Browning Strikeforce HD Pro, to avoid issues with intermodal differences (Apps & McNutt, 2018). Camera traps were mounted at shoulder height of the smallest species (60–80 cm high, the approximate shoulder height of the roe deer; The Mammal Society, 2022) with no angled dip downwards; placing cameras too high or dipping the field of view risks missing target species (Apps & McNutt, 2018; Palencia et al., 2022). Where possible, the immediate field of view of the camera trap was cleared of vegetation to both improve and standardise the reliability of detecting the target species and to reduce the probability of vegetation erroneously triggering the camera. Similarly, for camera traps positioned on slopes, the camera field of view was positioned perpendicular to the slope, so as to not restrict the view and therefore detection range. As the target ungulates are reasonably common onsite, leaving camera traps at a single location for a longer period was deemed more appropriate than moving cameras to new locations part way through the survey period (Mackenzie & Royle, 2005). Camera traps were programmed with a 1‐s delay between images. Having a 1‐s delay allowed for sequences of the same animal to be identified without being restricted to a pre‐defined number of images. 64 GB SD cards were used and image quality was set to 4 MB to ensure SD cards did not run out of memory prematurely. Both the motion detection and infra‐red flash were set as ‘long range’ to improve detectability.

### 2.3 Image tagging

Images were manually tagged with the species present using ExifPro v2.1 (Kowalski, 2013). Individuals present in the images were identified to species level. Then, following commonly used camera trapping methodology, the first image of a sequence of images of an individual animal, or images of the same species taken greater than 30 min apart, was tagged as independent ‘contacts’ (Mori et al., 2020; Ridout & Linkie, 2009; Sollmann et al., 2013; Zanni et al., 2021). Only these contacts were used in future analyses to reduce temporal correlation and increase independence, should one individual animal or group of individuals spend an extended period in front of the camera (Mori et al., 2020; Ridout & Linkie, 2009; Sollmann et al., 2013; Zanni et al., 2021). The same procedure was used for human contacts, with a human detection rate (human contacts per day) obtained for each camera trap.

**FIGURE 2:**
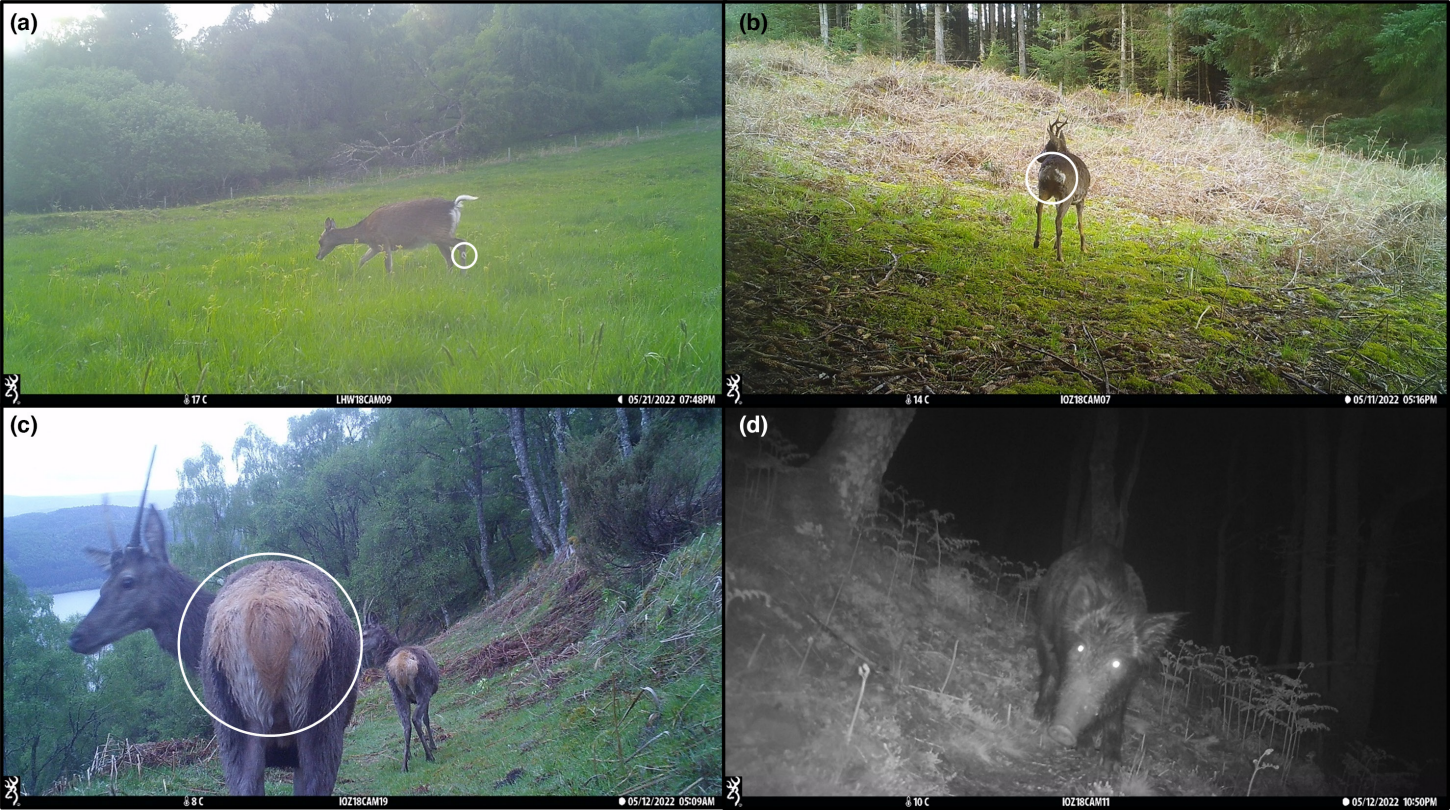
Camera trap photographs demonstrating all four target ungulate species (a = sika deer; b = roe deer; c = red deer; d = wild boar), with key identifying characters for the three deer species highlighted (sika deer = the white metatarsal gland; roe deer = the rump pattern and lack of obvious tail; red deer = the creamy, beige rump).

For species identification and tagging, we used these key diagnostic features (Figure 2; Couzens et al., 2021; The Mammal Society, 2022):

- The red deer is the largest species with the most complex antlers (up to eight points per antler) and has a creamy rump with a short reddish tail.
- The sika deer is the next largest species and has much simpler and thinner antlers than the red deer (with up to four points). The rump is white with a dark brown/black rim with a white tail featuring a single vertical stripe. An additional identification characteristic is a distinct white metatarsal gland on the lower hind legs.
- The roe deer is the smallest of the three species, with much smaller antlers (up to three points). They additionally have a broad, whitish rump either shaped as an inverted heart (females) or an oval (males) with no obvious tail.

### 2.4 Land cover classifications

From 2020 to 2021, multiple ecological surveys were conducted onsite as part of baseline biological surveys. In 2020 land cover onsite was classified into six different categories based on UK habitat classifications: clearfell, coniferous plantation, grassland, heathland and shrubland, wetland, woodland (Butcher et al., 2020; Highlands Rewilding Ltd, 2021a). These categories were confirmed with NVC survey data from 2021. Each camera trap site was assigned to one of these six land cover types via QGIS v.3.16.4 (QGIS Development Team, 2022) depending on the camera trap's location.

### 2.5 Statistical analysis

Data manipulation and statistical analyses were conducted using R v.4.2.2 (R Core Team, 2022). Results were deemed significant if *p*‐value <.05.

#### 2.5.1 Land cover preference modelling

Four negative binomial generalised linear models (GLM) were constructed to model the impact of land cover on ungulate detection rates across the camera traps. Negative binomial models used as overdispersion were detected in all models using performance v.0.10.2 (Lüdecke et al., 2021). *Species‐specific detection rate* acted as the response variable: with one GLM for each of the four ungulate species. *Land cover* and *human detection rate* acted as predictor variables, whilst the log of the *duration* each camera trap was deployed for acted as an offset variable to control for varying camera trap deployment times (i.e., to accommodate cases where a camera failed part way through; Sollmann, 2018). For the sika deer and roe deer, negative binomial GLMs were constructed using lme4 v.1.1‐29 (Bates et al., 2015), whilst for red deer and wild boar, negative binomial GLMs were constructed via glmmTMB v.1.1.6 (Brooks et al., 2017) to overcome issues with complete separation of the data. Finally, post‐hoc comparisons of estimated marginal means for each land cover type were undertaken via emmeans v.1.5.5‐1 (Lenth, 2021). Estimated marginal means (also known as least‐squares means) are the modelled means for each variable in the model, whilst accounting for other variables in the model (Lenth, 2021). Thus, here the estimated marginal means represent the predicted ungulate detection rate per day for each land cover class. Associated tables were constructed with sjPlot v.2.8.12 (Lüdecke, 2021) and figures with emmeans.

#### 2.5.2 Time interval modelling

Following Niedballa et al. (2019), we investigated whether an initial species arriving at a camera trap site (the leading species) affects the time until a second species (the follower species) is detected. As we calculated the time interval gaps on an individual camera trap basis, we incorporated both a spatial and temporal aspect, thereby undertaking a spatiotemporal analysis (Cusack et al., 2017; Niedballa et al., 2019).

For every species pair, we calculated the median time interval between the leading species arriving at a camera trap site and the follower species arriving, giving 12 observed median time intervals. Each observed median time interval was then compared to a null distribution of time intervals to identify if the follower species is attracted to or avoids the leading species. To create the null distribution for each species pair, each detection of the follower species was first assigned a random date, sampled from the dates that camera trap was deployed, and a random time, sampled from the daily activity pattern of the follower species. This generated a null dataset from which a new, simulated median was calculated. This was repeated 1000 times to finally generate the null distribution of simulated time intervals, representing the distribution of time intervals expected if the leading species does not influence the follower species (Niedballa et al., 2019).

The *p*‐value for the test was then calculated through a two‐sided significance test, where *Q* represents the proportion of null medians greater than the observed median (Niedballa et al., 2019):

$$p=\min\left(Q,1–Q\right)\times 2$$

## 3 RESULTS

One camera trap was faulty, and four other camera traps failed to record for the full length of time, leaving 1099 camera trap days successfully recorded. All four target ungulates were captured on camera traps (Table 1). On average it took 1.70 days to photograph a sika deer, 8.59 days to photograph a roe deer, 23.38 days to photograph a red deer, and 9.47 days to photograph a wild boar. 29 ungulate contacts were unable to be identified from camera trap images and were excluded, representing under 3% of all ungulate contacts.

**TABLE 1:** Summary statistics for the four ungulates recorded.

Species	Naïve occupancy (%)	Total number of contacts	Mean detection rate ± standard deviation
Sika deer	89.74	647	0.60 ± 0.92
Roe deer	74.36	128	0.12 ± 0.16
Red deer	53.85	47	0.04 ± 0.06
Wild boar	43.59	116	0.11 ± 0.20

*Note*: Naïve occupancy represents the proportion of camera traps where each species was detected at least once, total number of contacts records the total number of independent contacts of each species across all camera traps, and mean detection rate represents the mean number of independent detections per day for each species across camera traps ± the standard deviation.

### 3.1 Land cover preferences

Of the 39 successful camera traps, 2 were placed in clearfell, 8 in coniferous plantations, 8 in grassland, 6 in heathland and shrubland, 5 in wetland, and 10 in woodland.

Compared to clearfell, the detection rate of sika deer increased 11.94 times in coniferous plantations (estimate = 2.48, error = 1.07, *p* = .020), 28.22 times in grassland (estimate = 3.34, error = 1.07, *p* = .002), and 13.07 times in woodland (estimate = 2.57, error = 1.06, *p* = .015). Post‐hoc pairwise comparisons also detected elevated detection rates in grassland relative to heathland and shrubland (estimate = 1.72, error = 0.65, *p* = .008) and wetland (estimate = 1.74, error = 0.62, *p* = .005). Finally, sika deer detection rate increased with human detection rate (Table 2; Figure 1; estimate = 0.48, error = 0.18, *p* = .007).

**TABLE 2:** Outputs from the four GLMs investigating how land cover and human disturbance impacts sika deer, roe deer, red deer, and wild boar detection rates.

Predictors	Estimate	Std. error	*p*‐Value
Sika deer			
Clearfell (Intercept)	−3.41	1.00	**.001**
Coniferous plantation	2.48	1.07	**.020**
Grassland	3.34	1.07	**.002**
Heathland and shrubland	1.61	1.14	.157
Wetland	1.60	1.12	.156
Woodland	2.57	1.06	**.015**
Human detection rate	0.48	0.18	**.007**
*R* ^2^	.56		
Roe deer			
Clearfell (Intercept)	−2.97	0.90	**.001**
Coniferous plantation	1.16	0.98	.235
Grassland	1.09	0.98	.263
Heathland and shrubland	0.27	1.08	.800
Wetland	0.92	1.03	.373
Woodland	0.00	0.99	.998
Human detection rate	0.21	0.19	.249
*R* ^2^	.24		
Red deer			
Clearfell (Intercept)	−3.58	1.10	**.001**
Coniferous plantation	0.45	1.18	.703
Grassland	0.32	1.14	.782
Heathland and shrubland	0.33	1.21	.783
Wetland	1.33	1.15	.245
Woodland	−0.15	1.18	.902
Human detection rate	0.21	0.16	.169
*R* ^2^	.07		
Wild boar			
Clearfell (Intercept)	−6.36	3.40	.061
Coniferous plantation	0.86	3.57	.810
Grassland	5.10	3.42	.136
Heathland and shrubland	2.61	3.49	.454
Wetland	−5.99	44.56	.893
Woodland	4.43	3.42	.196
Human detection rate	0.09	0.26	.737
*R* ^2^	.84		

*Note*: Significant *p*‐values are highlighted in bold.

**FIGURE 3:**
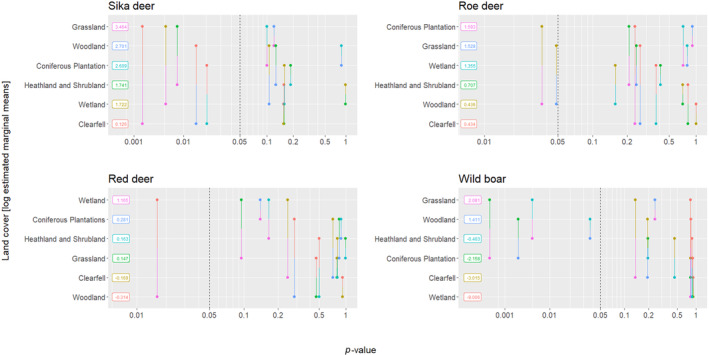
Four pairwise *p*‐value plots demonstrating the pairwise comparisons between different land cover classes for each ungulate species. Land cover classes (with the log estimated marginal means present in boxes) are on the *y*‐axis, with vertical lines between land cover classes representing each pairwise comparisons. The positioning of the vertical comparison along the *x*‐axis indicates its associated *p*‐value. Comparisons to the left of the vertical dashed line (*p* = .05) are deemed significant.

Land cover was not initially found to significantly predict roe deer detection rate. However, post‐hoc pairwise comparisons detected lower detection rates in woodland compared to both coniferous plantations (estimate = 1.16, error = 0.55, *p* = .036) and grassland (Table 2; Figure 3; estimate = 1.09, error = 0.55, *p* = .049).

Similarly, land cover was not initially found to significantly predict red deer detection rate. However, post‐hoc pairwise comparisons detected higher detection rates in wetland relative to woodland (Table 2; Figure 3; estimate = 1.48, error = 0.62, *p* = .016).

Finally, land cover was also not initially found to significantly predict wild boar detection rate. However, post‐hoc pairwise comparisons detected higher detection rates in grassland relative to coniferous plantation (estimate = −4.24, error = 1.18, *p* < .001) and heathland and shrubland (estimate = 2.48, error = 0.88, *p* = .005). Similarly, higher detection rates were present in woodland compared to coniferous plantation (estimate = −3.57, error = 1.18, *p* = .003) and heathland and shrubland (Table 2; Figure 3; estimate = −1.81, error = 0.87, *p* = .037).

### 3.2 Time interval modelling

**FIGURE 4:**
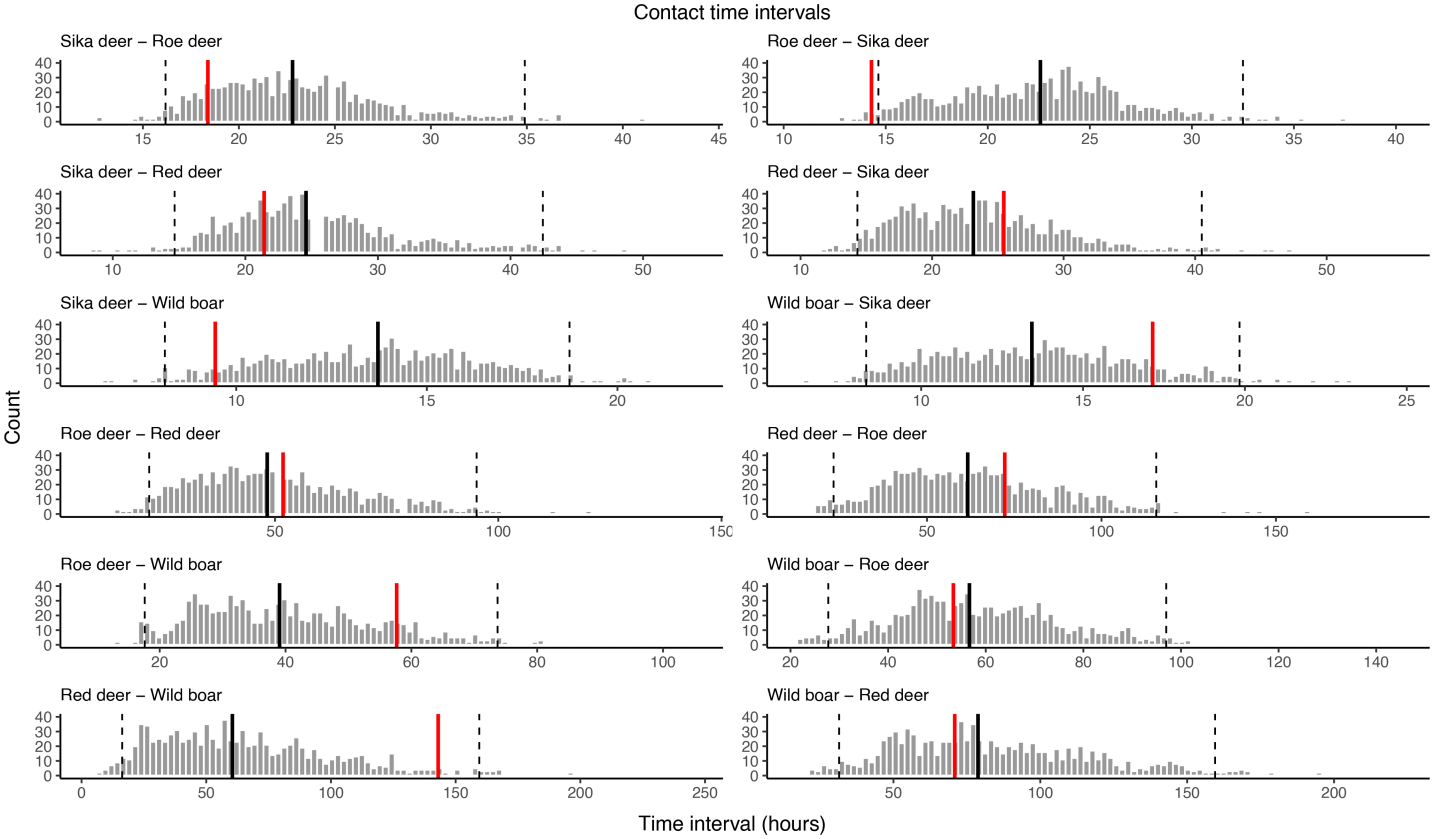
Outputs from the time interval modelling approach. Each plot represents the results from one pairwise interaction. For example, Sika deer–Roe deer represents the time differences, both observed and expected, between a leading sika deer arriving at a camera trap and a roe deer follower arriving at a camera trap. For each tested interaction, the red vertical line represents the observed median time interval, the black vertical line represents the modelled median time interval, and grey vertical bars represent the distribution of 1000 modelled null time intervals. Dashed vertical lines represent the 95% confidence intervals for attraction and avoidance.

Time interval modelling detected an attractive effect between roe deer and sika deer. The time interval between a roe deer arriving at a camera trap site and a sika deer following was approximately 36.7% shorter than would be expected from no effect (Figure 4b; *p* = .038). No other relationships were statistically significant (Figure 4a,c–l).

## 4 DISCUSSION

Using a camera trap survey, we quantified land cover preferences, the influence of human disturbance, and spatiotemporal relationships between ungulate species within a unique mammal community in Scotland. As expected, land cover was found to play a role in dictating ungulate detection rates. Although land cover preferences were detected, the GLMs constructed for roe and red deer had low *R* ^2^ values, limiting the explanatory value of these models. In addition, whilst the 511 ha site represents an area greater than the largest average summer home range estimates recorded in studies of the sika deer (400 ha; Borkowski & Furubayashi, 1998; Laneng et al., 2023; Putman & Pemberton, 2022), roe deer (140 ha; Lovari et al., 2017, Mysterud, 1998, Pagon et al., 2017), and wild boar (420 ha; Keuling et al., 2008; Podgórski et al., 2013; Russo et al., 1997), it is smaller than the typical range size of the red deer, and thus the identified land cover preferences for red deer should be interpreted with caution (Catt & Staines, 1987; Kamler et al., 2008). We found no evidence of a negative effect of human disturbance on ungulate distribution, instead finding a positive effect of human detection rate on sika deer. Finally, although we had anticipated competition to drive spatiotemporal avoidance, we found no evidence of this. Instead, a spatiotemporal association of sika deer for roe deer was detected.

This study being conducted in spring will likely impact land cover preferences and spatiotemporal interactions. In spring, the ungulates present will feed on new vegetation growth to maximise energy intake after harsher winters, with ungulates heavily pregnant, birthing, and feeding young at this time of year (Gaillard et al., 1993; McCullough et al., 2009; Pettorelli, Gaillard et al., 2003; Stopher et al., 2008). Roe deer are additionally defending territories in May/June until late August, after the rut ends (Johansson, 1996). As income breeders, roe deer need to feed to replace energy lost during this high‐activity period, meaning any preferences for high‐quality forage are likely strongest at this time of year (Pagon et al., 2017).

For sika deer, the strong preference for grassland likely reflects grazing. Although grasslands potentially bias detection rates due to the lack of vegetation, sika deer act as mostly grazers in both their native and introduced ranges (Endo et al., 2017; Putman, 1996; Putman & Pemberton, 2022; Sollmann, 2018). Indeed, sika deer are known to move out of cover onto adjacent open ground and heath at night to feed (Putman, 1996). In addition, prior work identifies a preference for coniferous plantations and woodland – similar to our results (Mayle, 1996; Putman, 1996; Putman & Pemberton, 2022; Sakuragi et al., 2002). Indeed, mixed woodland and coniferous forest are highly used vegetation types of sika deer in Japan (Sakuragi et al., 2003). Sika deer eat both deciduous and coniferous trees; hence, their presence in mixed woodland and coniferous forests could be due to feeding, whilst also exploiting the cover provided by the former two food sources (Akashi & Terazawa, 2005; Latham, 2000; Putman & Pemberton, 2022; Yokoyama et al., 2001).

Like sika deer, grasslands can provide forage for wild boar, potentially explaining their preference for grassland. Due to their rooting behaviour, wild boar turn over the soil and consume high quantities of underground plant parts and roots, with smaller, but consistent, quantities of invertebrates also foraged (Ballari & Barrios‐García, 2014; Endo et al., 2017; Schley & Roper, 2003). Furthermore, graminoids have been identified as a staple food resource for wild boar, with wild boar found to graze more over spring–summer time, when this study was conducted (Genov, 1981; Massei et al., 1996; Sandom et al., 2013a). The preference of wild boar for woodland over coniferous plantations is supported by studies outside of the UK, despite *Pinus* sp. providing a high‐quality fat resource (Fonseca, 2008; Liu et al., 2022; Muthoka et al., 2022; van Ginkel et al., 2013). As *Quercus* species were found throughout the woodland, it seems likely wild boar were active here to forage and root for acorns, in addition to bracken (*Pteridium aquilinum*) rhizomes, whilst deriving additional benefit from the cover provided by the woodland environment (Herrero et al., 2005; Highlands Rewilding Ltd, 2021a; Sandom et al., 2013a; van Ginkel et al., 2013). This study additionally adds to previous work from Sandom et al. (2013a) by demonstrating that wild boar in Scotland do utilise woodland when allowed to roam freely.

For roe deer, land cover preferences were less clear, with woodland only identified as avoided compared to coniferous plantations and grassland. These less clear preferences may be attributable to roe deer selecting areas based on the presence of particular forbs or shrubs, rather than the macro‐scale vegetation characteristics studied here (Mancinelli et al., 2015; Pettorelli, Dray, et al., 2003). Roe deer may select coniferous plantations as they provide a high‐cover environment relative to other land cover classes, with roe deer also possibly browsing on pine saplings (Palmer & Truscott, 2003). However, why this would lead to an avoidance of woodland is less clear, as roe deer also require woodland and forest strands with a richer understory (Lovari et al., 2017; Mancinelli et al., 2015). Despite roe deer being termed a ‘forest ungulate’, studies highlight that roe deer are adapted for more open grasslands and glades, and require just a minimum quantity of woodland within their home ranges (Lovari et al., 2017; Morellet et al., 2011). Roe deer may additionally be avoiding wooded areas due to the presence of wild boar, as seen in similar studies, although no significant spatiotemporal avoidance was detected in this study system (Zanni et al., 2021).

Similarly, red deer have less clear land cover preferences, with only a preference for wetland detected over woodland. Red deer are known to exploit both wet and dry open heathland, which may partially explain these results as much onsite wetland is dominated by species such as *Calluna vulgaris* and *Erica* sp. (Pérez‐Barbería et al., 2013; Plantlife, 2021; Putman, 1996; Ward, 2005; Welch et al., 1990). In addition, the largest area of wetland on the Bunloit estate runs alongside the North‐West of the site and directly borders an area where little‐to‐no deer management takes place, which itself is surrounded by deer stalking estates in the wider area which maintain large red deer herds (Highlands Rewilding Ltd, 2021b). The preference for wetland over woodland is in line with other studies, where red deer are found to avoid dense woodlands and forests except where there are clearings and glades (Mitchell et al., 1977; Welch et al., 1990). Although some level of woodland or forest cover does appear important for red deer elsewhere, red deer in Scotland are observed to have stronger preferences for open wetland and moorland over woodlands and forests (Borowik et al., 2013; Mitchell et al., 1977). Mitchell et al. (1977) suggest that open moorland and wetland in Scotland provides red deer with important habitat features which are provided by woodland elsewhere. For example, browse in the form of heather (*Calluna vulgaris*) is present, and unrestricted views may give the same sense of security that woodland can provide (Mitchell et al., 1977).

As keystone species, these ungulate species considered in this study are likely to impact the ecosystems they are found in through their land cover preferences. Sika deer, wild boar, and roe deer all had some preference for grassland, hypothesised here to be representative of grazing, with the former two also preferring woodland, hypothesised to be providing a mix of forage and cover. By altering the importance of stochastic and deterministic processes, ungulate grazing and rooting within these preferred land covers alter the species and functional diversity and composition of plants (Cushman et al., 2004; Nishizawa et al., 2016; Ohashi & Hoshino, 2014; Wardle et al., 2004). Even if not feeding, other impacts of ungulates such as trampling can impact plant communities (Barros & Pickering, 2015; Heggenes et al., 2017). These changes to functional diversity and composition can subsequently influence ecosystem processes and functions, such as carbon storage and seedling recruitment (Allen et al., 2023; Velamazán et al., 2020; Wardle et al., 2004). Over long time periods, these changes can significantly alter ecosystems. In woodlands, ungulates may inhibit tree regeneration and reduce fire risk, whilst in grassland soil carbon stocks may be significantly altered depending on the local conditions (Cornelissen et al., 2014; Lecomte et al., 2019; McSherry & Ritchie, 2013).

Contrary to our hypothesis, we detected no negative impact of human disturbance on ungulates, and instead detected a positive relationship between human and sika deer distribution. These results were unexpected as prior studies show ungulates avoiding areas of high human activity (Bojarska et al., 2020; Bonnot et al., 2013; D'Amico et al., 2016; Ikeda, Kuninaga et al., 2019). The observed preferences for high‐cover vegetation offering seclusion from human activity may mask any impact of human disturbance (Wevers et al., 2020). Indeed, Jayakody et al. (2008) found red deer vigilance behaviour was unaffected by human disturbance in high‐cover vegetation, such as woodland. Alternatively, the primarily diurnal activity of humans may complement a more crepuscular or nocturnal ungulate activity, allowing ungulates to coexist alongside human activity (Akbaba & Ayaş, 2012; Ikeda, Kuninaga et al., 2019; Ikeda, Takahashi et al., 2019). For sika deer, previous studies demonstrate behavioural flexibility in response to perceived predation risk through culling (Ikeda, Takahashi et al., 2019; Ikeda & Koizumi, 2024). Thus, human disturbance may not impact ungulate distributions if they are able to avoid high‐disturbance areas during daytime hours.

To our knowledge, this is the first time that spatiotemporal relationships among this community of ungulate species have been investigated. Unlike this study, most previous research does not explicitly consider both spatial and temporal aspects simultaneously in their analyses, either considering them separately, or aggregating hourly data across days (Melberg, 2012; Mori et al., 2020; Zanni et al., 2021). These approaches could make previous spatiotemporal relationships detected between species less reliable (Cusack et al., 2017).

No spatiotemporal avoidance was detected between any of the four species. This was contrary to our initial hypothesis, which predicted spatiotemporal avoidance would be present, although some studies have detected attraction and facilitation between ungulates. Bartos et al. (2002) found a lack of antagonistic interactions between ungulate species including roe deer, whilst Imperio et al. (2012) more specifically found both red deer and wild boar to be positively influenced by roe deer. Having said this, these particular interactions were not recovered in this study. One possible explanation for the lack of apparent spatiotemporal avoidance could be dietary partitioning. Wild boar typically feed less on grasses and woody browse than deer, instead opportunistically feeding on fruits/seeds, underground plant parts, and non‐plant items (Ballari & Barrios‐García, 2014; Schley & Roper, 2003; Spitzer et al., 2020). The three remaining deer species may split their foraging behaviour along a ‘browser–grazer axis’, with the smaller roe deer acting as a ‘browser’ by selecting smaller amounts of the most nutritious food items and the larger red and sika deer acting more as ‘grazers’ and able to consume larger amounts of low‐quality, fibrous forage (Endo et al., 2017; Putman & Pemberton, 2022; Spitzer et al., 2020; Storms et al., 2008). How the sika deer and red deer would differentiate is less certain, but both species can demonstrate large variation in forage types, with red deer in particular suggested to act more as an intermediate feeder (Endo et al., 2017; Fraser, 1996; Zhong et al., 2020). Finally, interactions between two ungulate species are more plastic than is typically assumed, and the interaction type can vary depending on factors such as an individual's life stage, the time of year, and environmental factors (Bronstein, 1994; Ferretti & Fattorini, 2021; Thompson, 1988). If sufficient resources are found onsite, then hypothesised competition between species may be reduced, leading to the interspecific interactions between ungulates manifesting non‐competitively (Ferretti & Fattorini, 2021).

A significant spatiotemporal association was detected for sika deer towards roe deer. This observed spatiotemporal association may indicate beneficial facilitatory relationships between the sika deer and roe deer (Asefa, 2016). Spatiotemporal associations could be driven by a combination of grazing facilitation and an anti‐predator response. Grazing facilitation is where, through feeding, one species makes forage in an area more accessible or preferable to a second species (Colman et al., 2009; Gordon, 1988; Odadi et al., 2011). As sika deer and roe deer diets split along a grazing‐browsing axis, the browsing action of the roe deer may be making grasses more accessible to sika deer (Mann, 1982; Putman, 1996; Putman & Pemberton, 2022; Spitzer et al., 2020; Tixier & Duncan, 1996). Secondly, although no large wild predators still reside in Scotland, the culling programme on the Bunloit rewilding project could be driving an anti‐predator response and spatiotemporal associations between ungulates, with the heavily culled sika deer at the centre of this (Highlands Rewilding Ltd, 2021a, 2021b). Indeed, sika deer are known to shift their activity patterns in response to culling, with an increase in nocturnal activity observed, whilst ungulates can form interspecific groups as an anti‐predator response (Asefa, 2016; Bartos et al., 2002; Ikeda, Takahashi et al., 2019). Despite these mutually inclusive suggestions, more research is required to identify the underlying mechanisms behind these spatiotemporal associations.

Camera traps are a successful way to remotely monitor wild animal populations in a less invasive and biased manner than traditional transect approaches (Marini et al., 2009). However, camera trapping is not without its own limitations. For starters, a species is only recorded as present when it is successfully detected and photographed by a camera trap. However, whether a species is successfully detected by a camera trap is partially dependent on camera trap‐level variables, such as the density of vegetation or the field of view, which could risk biasing results (Sollmann, 2018). Furthermore, although bias between camera traps was reduced by limiting the study to one site over a single season, with a consistent camera trap type and set‐up, there may be differences in detectability between species (Hofmeester et al., 2019). However, failure for a camera trap to trigger appears to be less of a problem for large‐bodied species such as the ungulates studied here (Kays et al., 2021). Despite being less invasive than other techniques such as telemetry and nocturnal transects, camera traps can be noticed by wildlife and can trigger a behavioural reaction, with both attraction to and avoidance of camera traps observed (Henrich et al., 2020; Marini et al., 2009; Meek et al., 2014; Roberts, 2011). Operational limitations of camera traps can further hinder data collection, ranging from condensation distorting photographs to failure of camera traps (Newey et al., 2015).

With novel land uses and ungulate communities present the Scottish Highlands, it remains important to understand the dynamics of ungulate communities. Using a camera trapping survey, this study provided a snapshot understanding of the spring–summer land cover preferences of sika deer, red deer, roe deer, and wild boar. For wild boar, this is one of the first studies assessing its land cover preferences and activities in Scotland, with prior studies limited to captive populations over smaller areas (Sandom et al., 2013a, 2013b). As a keystone native species with only two to four current populations in Scotland, the formal reintroduction of wild boar to Scotland has been proposed (Leaper et al., 1999; Sandom et al., 2013a, 2013b). The results from this study highlights the strong preference of wild boar for grassland and woodland land cover types, and therefore could be used to inform any proposed wild boar reintroductions of the potential land cover preferences of released individuals and the permeability of the landscape. We additionally used spatiotemporal analyses to provide evidence of an attractive effect of sika deer towards roe deer. To our knowledge, this is the first study to identify this spatiotemporal association, and further research is needed to both confirm this effect and understand the underlying reason for this. Should roe deer partially influence sika deer activity, then any changes in population size of roe deer (for example, through culling) could lead to unanticipated changes to sika deer activity and thus environmental impacts, something which land managers and conservation organisations should consider.

## AUTHOR CONTRIBUTIONS

**Connor Lovell:** Conceptualization (lead); data curation (lead); formal analysis (lead); methodology (lead); visualization (lead); writing – original draft (lead); writing – review and editing (lead). **Nathalie Pettorelli:** Conceptualization (supporting); formal analysis (supporting); methodology (supporting); supervision (equal); writing – review and editing (equal). **Terence P. Dawson:** Conceptualization (supporting); formal analysis (supporting); methodology (supporting); supervision (equal); writing – review and editing (equal).

## CONFLICT OF INTEREST STATEMENT

The authors have no competing interests to declare that are relevant to the content of this article.

## Data Availability

The datasets generated and analysed during the current study are available at https://datadryad.org/stash/share/WfDzjay4hwuCllPeOljPT_zL3dbUq6Thdg1arBVOz9Q.
